# Improving Water and Nutrient Use Efficiency in Rice by Changing Crop Establishment Methods, Application of Microbial Inoculations, and Zn Fertilization

**DOI:** 10.1002/gch2.201800005

**Published:** 2019-02-06

**Authors:** Amit Anil Shahane, Yashbir Singh Shivay, Radha Prasanna, Dinesh Kumar

**Affiliations:** ^1^ Division of Agronomy ICAR—Indian Agricultural Research Institute New Delhi 110012 India; ^2^ Division of Microbiology ICAR—Indian Agricultural Research Institute New Delhi 110012 India

**Keywords:** aerobic rice system, microbial inoculants, system of rice intensification, water productivity, zinc fertilization

## Abstract

A field experiment was conducted during the wet seasons of 2013 and 2014 to evaluate the effect of three rice establishment methods: viz. puddled transplanted rice (PTR), system of rice intensification (SRI) and aerobic rice system (ARS), two cyanobacteria based inoculants, viz. *Anabaena sp* (CR1) + *Providencia sp* (PR3) consortium and *Anabaena–Pseudomonas* (An‐Ps) biofilm formulation, and zinc (Zn) fertilization on the rice yield, water productivity, and nutrient use efficiency. The yields of rough, brown and milled rice were highest in SRI, which was on par with PTR and both methods proved significantly superior to ARS in both years. The total water productivity of rough and brown rice in the first year was significantly higher in SRI. The SRI method saved 21.9% and 27.4% irrigation water over PTR, and savings in ARS were 37.4% and 50.8% in first and second year respectively, over PTR. The use of An‐Ps biofilm formulation along with 75% RDN improved the agronomic use efficiency of both nitrogenous and phosphatic fertilizers applied. On the basis of the present study, it can be concluded that SRI improved rice yields and water productivity; while involvement of An‐Ps biofilm formulation can be recommended for improved nutrient use efficiency.

## 1 Introduction

Rice is the staple food in south and southeast Asia and plays an important role in Indian agriculture, as it occupies the largest area (43.5 million ha) among the field crops and also stands first in production (104.3 million tonnes). The economic importance of rice ecosystems in the world can be judged from its coverage of 163 million ha with a production of 741 million tonnes of grain, ranking third in area and second in production among all the arable crops.^1^ Rice crop accounts for 37% of the nitrogen and phosphate fertilizers' consumption,^2^ besides 22% of the pesticides used in India.^3^ Rice is a major consumer of agricultural inputs in India, including water. Based on hydrological regimes, soils, and climatic conditions, rice production systems are classified into six different categories,^4^ namely, irrigated (wet season), irrigated (dry season), rain‐fed upland, rain‐fed lowland, deep water, and coastal lowland production systems. The irrigated rice production system covers about 44%, while rain‐fed production system covers about 45% of the global rice area. The availability of water, although considered a renewable natural resource, is greatly dependent upon the productivity of the source (river, canal, and pond) and the distribution system prevalent in a region/country. In the case of groundwater as the source, availability of water is governed by the availability of electricity. Quality of water is also a concern in some regions. Adequate water availability and its easy access are major factors determining rice yields and several researchers have quantified its requirement^5^, ^6^ and share in the total available fresh water.^7^ Serious concern has been raised regarding the depletion of surface water resources, reducing levels of groundwater, and lowering of water table due to rice cultivation in India.^8^, ^9^, ^10^, ^11^ The major methods of rice cultivation—puddled transplanted rice (PTR), aerobic rice system (ARS),^12^ and system of rice intensification (SRI)—differ in their water requirements.^13^ A comparative study is needed for assessing their potential in terms of water savings. In the era of climate change, water availability is critical,^14^, ^15^ and with growing population‐driven demand for rice,^16^, ^17^ evaluating different cultivation methods for their influence on rice yield and water saving potential is an important agenda.

Along with water, fertility status and nutrient supplying capacity of soil in rice growing area need due attention. The depletion of soil fertility and zinc deficiency are major problems in rice–wheat cropping system, particularly in India.^18^ Among the nutrients, nitrogen (N) is universally deficient in soil and recovery of applied N is usually less than 50%,^19^ while phosphorus (P) is receiving more attention as a nonrenewable resource^20^ with a low availability due to slow diffusion and high fixation in soils.^21^ Zinc deficiency ranks fifth among the most important health risk factors in developing countries^22^ and proper Zn management contributes about 18.4 million tonnes of grains (211.6 billion) for major food grain crops.^23^ Interactions between the root and the root microbiome influence plant growth as several microorganisms in the rhizosphere share a symbiotic or associative relationship with the plant.^24^ The use of microbial inoculation in the nutrient management of rice is known to elicit positive effects on growth, yield, and nutrient uptake. Among microbes, the role of cyanobacteria and their consortium/biofilms with agriculturally beneficial bacteria in improving plant growth by N fixation and solubilizing soil P has been reported,^25^, ^26^, ^27^, ^28^ and its promise is well established in conventional puddled transplanted rice system, besides recent reports on their promise in SRI, but not in aerobic rice.^28^, ^29^ However, no information is available regarding the comparative efficacy of these inoculants in SRI and ARS, vis‐à‐vis PTR, in terms of improving yield and water savings; with this background, this study was undertaken. The information generated can be valuable not only for researchers, but also for policy makers.

## 2 Results

### 2.1 Rice Yields

The yields of rough, brown, and milled rice were higher during the first year, as compared to those in the second year (**Tables** **1**–**3**). The SRI method recorded the highest yield of rough rice in both the years and remained at par with PTR, while both PTR and SRI performed significantly better than the ARS during both the years of study (Table 1). The application of recommended dose of nutrients (RDN) + Zn recorded the highest rough rice yield, which was significantly superior to the application of RDN in all crop establishment methods (CEMs). The yields in two treatments involving microbial inoculants, namely, 75% RDN + *Anabaena*–*Pseudomonas* (An–Ps) biofilm formulation and 75% RDN + *Anabaena* sp. (CR1) + *Providencia* sp. (PR3), were at par with each other and with the RDN in all CEMs.

**Table 1:** Influence of crop establishment methods and nutrient management options on rough rice yield during 2013 and 2014

Treatment^a)^	Control (N_0_P_0_Zn_0_)	100% RDN	100% RDN^b)^ + Zn^c)^	75% RDN	75% RDN + Zn	75% RDN + CR1 + PR3^d)^	75% RDN + CR1 + PR3^d)^ + Zn	75% RDN + An–Ps^e)^ biofilm formulation	75% RDN + An–Ps^e)^ biofilm formulation + Zn	Mean
Rough rice yield during 2013 (Mg ha^−1^)										
PTR	3.23	4.21	4.36	3.87	4.03	4.18	4.34	4.18	4.33	4.08
SRI	3.40	4.20	4.35	3.87	4.03	4.17	4.32	4.16	4.35	4.10
ARS	3.21	4.06	4.18	3.71	3.90	4.00	4.16	4.00	4.15	3.93
Mean	3.28	4.16	4.30	3.82	3.99	4.12	4.27	4.12	4.28	
			Systems of cultivation	Nutrient management options	Interaction					
		SEM	0.02	0.03	0.05					
		CD (*P* = 0.05)	0.06	0.09	0.15					
Rough rice yield during 2014 (Mg ha^−1^)										
PTR	3.05	4.06	4.28	3.82	3.89	4.01	4.23	4.03	4.22	3.95
SRI	3.22	4.04	4.28	3.81	3.89	4.01	4.22	4.02	4.24	3.97
ARS	3.03	3.90	4.10	3.65	3.75	3.83	4.06	3.86	4.05	3.80
Mean	3.10	4.00	4.22	3.76	3.84	3.95	4.17	3.97	4.17	
			Systems of cultivation	Nutrient management options	Interaction					
		SEM	0.01	0.02	0.04					
		CD (*P* = 0.05)	0.04	0.06	0.11					

^a)^ PTR, puddled transplanted rice; SRI, system of rice intensification; ARS, aerobic rice system

^b)^ Recommended dose of nutrients (120 kg nitrogen ha^−1^ and 25.8 kg phosphorus ha^−1^)

^c)^ 5 kg Zn ha^−1^ through ZnSO_4_·7H_2_O

^d)^ *Anabaena* sp. (CR1) + *Providencia* sp. (PR3) consortium

^e)^ *Anabaena*–*Pseudomonas* biofilm formulation.

**Table 2:** Influence of crop establishment methods and nutrient management options on brown rice yield during 2013 and 2014

Treatment^a)^	Control (N_0_P_0_Zn_0_)	100% RDN	100% RDN^b)^ + Zn^c)^	75% RDN	75% RDN + Zn	75% RDN + CR1 + PR3^d)^	75% RDN + CR1 + PR3^d)^ + Zn	75% RDN + An–Ps^e)^ biofilm formulation	75% RDN + An–Ps^e)^ biofilm formulation + Zn	Mean
Brown rice yield during 2013 (Mg ha^−1^)										
PTR	2.40	3.31	3.41	3.01	3.10	3.27	3.40	3.27	3.38	3.17
SRI	2.56	3.32	3.45	2.96	3.11	3.26	3.40	3.31	3.43	3.20
ARS	2.38	3.09	3.23	2.79	2.95	3.07	3.20	3.03	3.23	3.00
Mean	2.45	3.24	3.36	2.92	3.05	3.20	3.34	3.20	3.35	
			Systems of cultivation	Nutrient management options	Interaction					
		SEM	0.01	0.02	0.04					
		CD (*P* = 0.05)	0.05	0.06	0.10					
Brown rice yield during 2014 (Mg ha^−1^)										
PTR	2.26	3.17	3.33	2.93	2.97	3.12	3.30	3.14	3.27	3.05
SRI	2.41	3.18	3.37	2.89	2.97	3.11	3.30	3.18	3.32	3.08
ARS	2.23	2.95	3.15	2.72	2.82	2.92	3.11	2.90	3.12	2.88
Mean	2.30	3.10	3.28	2.85	2.92	3.05	3.24	3.07	3.24	
			Systems of cultivation	Nutrient management options	Interaction					
		SEM	0.01	0.02	0.03					
		CD (*P* = 0.05)	0.03	0.05	0.08					

^a)^ PTR, puddled transplanted rice; SRI, system of rice intensification; ARS, aerobic rice system

^b)^ Recommended dose of nutrients (120 kg nitrogen ha^−1^ and 25.8 kg phosphorus ha^−1^)

^c)^ 5 kg Zn ha^−1^ through ZnSO_4_·7H_2_O

^d)^ *Anabaena* sp. (CR1) + *Providencia* sp. (PR3) consortium

^e)^ *Anabaena*–*Pseudomonas* biofilm formulation.

**Table 3:** Influence of crop establishment methods and nutrient management options on milled rice yield during 2013 and 2014

Treatment^a)^	Control (N_0_P_0_Zn_0_)	100% RDN	100% RDN^b)^ + Zn^c)^	75% RDN	75% RDN + Zn	75% RDN + CR1 + PR3^d)^	75% RDN + CR1 + PR3^d)^ + Zn	75% RDN + An–Ps^e)^ biofilm formulation	75% RDN + An–Ps^e)^ biofilm formulation + Zn	Mean
Milled rice yield during 2013 (Mg ha^−1^)										
PTR	2.06	2.83	2.99	2.58	2.64	2.87	2.98	2.85	2.88	2.74
SRI	2.20	2.87	2.97	2.50	2.66	2.86	2.92	2.84	3.02	2.76
ARS	2.02	2.69	2.80	2.40	2.54	2.56	2.77	2.60	2.79	2.57
Mean	2.09	2.79	2.92	2.49	2.61	2.76	2.89	2.76	2.90	
			Systems of cultivation	Nutrient management options	Interaction					
		SEM	0.01	0.03	0.05					
		CD (*P* = 0.05)	0.06	0.08	0.14					
Milled rice yield during 2014 (Mg ha^−1^)										
PTR	1.90	2.67	2.86	2.48	2.48	2.69	2.83	2.68	2.74	2.59
SRI	2.04	2.70	2.85	2.40	2.50	2.68	2.73	2.68	2.87	2.61
ARS	1.87	2.53	2.67	2.30	2.38	2.39	2.70	2.45	2.65	2.44
Mean	1.94	2.63	2.79	2.40	2.46	2.59	2.76	2.60	2.75	
			Systems of cultivation	Nutrient management options	Interaction					
		SEM	0.01	0.02	0.03					
		CD (*P* = 0.05)	0.04	0.06	0.10					

^a)^ PTR, puddled transplanted rice; SRI, system of rice intensification; ARS, aerobic rice system

^b)^ Recommended dose of nutrients (120 kg nitrogen ha^−1^ and 25.8 kg phosphorus ha^−1^)

^c)^ 5 kg Zn ha^−1^ through ZnSO_4_·7H_2_O

^d)^ *Anabaena* sp. (CR1) + *Providencia* sp. (PR3) consortium

^e)^ *Anabaena–Pseudomonas* biofilm formulation.

The brown rice yield was less than rough rice by 910 kg ha^−1^ in PTR, 900 kg ha^−1^ in SRI, and 930 kg ha^−1^ in ARS during the first year and 900 kg ha^−1^ in PTR, 890 kg ha^−1^ in SRI, and 920 kg ha^−1^ in ARS during the second year of study. The yield of brown rice was 170 kg ha^−1^ higher in PTR and 200 kg ha^−1^ higher in SRI than ARS (Table 2). Three treatments containing RDN + Zn, 75% RDN + An–Ps biofilm formulation + Zn, and 75% RDN + CR1 + PR3 + Zn recorded significantly higher brown rice yield than similar treatments without zinc application in all CEMs; the yields of these three treatments were significantly higher than all other nutrient management options. The milled rice yield in PTR was 67.2% of rough rice and 86.4% of brown rice yield during the first year and 65.6% and 84.9%, respectively, during the second year (Table 3). Similarly, the yield of milled rice in SRI was 86.3% and 84.7% of brown rice and 65.3% and 65.7% of rough rice, during the first and second years, respectively. In the ARS method, percent contribution of rough rice to milled rice was lower than that in PTR and SRI, which was 65.4% and 64.2% during the first and second years, respectively. The highest bran yield was recorded in SRI and the highest hull yield in ARS in both the years (**Tables** **4** and **5**). The yields of bran and hull remained on par in all nutrient management treatments, except control, which showed significantly lower bran and hull yield than rest of the treatments in all CEMs. The highest bran yield was recorded in 75% RDN + CR1 + PR3 + Zn in the SRI method in the second year, while highest hull yield was recorded in 75% RDN + An–Ps biofilm formulation + Zn in ARS during the first year.

**Table 4:** Influence of crop establishment methods and nutrient management options on bran yield during 2013 and 2014

Treatment^a)^	Control (N_0_P_0_Zn_0_)	100% RDN	100% RDN^b)^ + Zn^c)^	75% RDN	75% RDN + Zn	75% RDN + CR1 + PR3^d)^	75% RDN + CR1 + PR3^d)^ + Zn	75% RDN + An–Ps^e)^ biofilm formulation	75% RDN + An–Ps^e)^ biofilm formulation + Zn	Mean
Bran yield during 2013 (Mg ha^−1^)										
PTR	0.343	0.475	0.428	0.426	0.461	0.396	0.422	0.420	0.503	0.430
SRI	0.364	0.455	0.474	0.460	0.445	0.405	0.484	0.469	0.409	0.441
ARS	0.361	0.398	0.431	0.393	0.417	0.504	0.434	0.424	0.438	0.422
Mean	0.356	0.443	0.444	0.426	0.441	0.435	0.447	0.438	0.450	
			Systems of cultivation	Nutrient management options	Interaction					
		SEM	0.01	0.02	0.03					
		CD (*P* = 0.05)	0.04	0.05	0.08					
Bran yield during 2014 (Mg ha^−1^)										
PTR	0.358	0.500	0.476	0.452	0.481	0.429	0.469	0.456	0.536	0.462
SRI	0.371	0.480	0.521	0.485	0.466	0.437	0.568	0.504	0.445	0.475
ARS	0.364	0.424	0.477	0.418	0.437	0.530	0.404	0.457	0.470	0.442
Mean	0.364	0.468	0.492	0.452	0.461	0.465	0.481	0.472	0.484	
			Systems of cultivation	Nutrient management options	Interaction					
		SEM	0.01	0.01	0.02					
		CD (*P* = 0.05)	0.04	0.04	0.07					

^a)^ PTR, puddled transplanted rice; SRI, system of rice intensification; ARS, aerobic rice system

^b)^ Recommended dose of nutrients (120 kg nitrogen ha^−1^ and 25.8 kg phosphorus ha^−1^)

^c)^ 5 kg Zn ha^−1^ through ZnSO_4_·7H_2_O

^d)^ *Anabaena* sp. (CR1) + *Providencia* sp. (PR3) consortium

^e)^ *Anabaena–Pseudomonas* biofilm formulation.

**Table 5:** Influence of crop establishment methods and nutrient management options on hull yield during 2013 and 2014

Treatment^a)^	Control (N_0_P_0_Zn_0_)	100% RDN	100% RDN^b)^ + Zn^c)^	75% RDN	75% RDN + Zn	75% RDN + CR1 + PR3^d)^	75% RDN + CR1 + PR3^d)^ + Zn	75% RDN + An–Ps^e)^ biofilm formulation	75% RDN + An–Ps^e)^ biofilm formulation + Zn	Mean
Hull yield during 2013 (Mg ha^−1^)										
PTR	0.82	0.91	0.94	0.87	0.93	0.91	0.93	0.91	0.95	0.91
SRI	0.84	0.88	0.91	0.91	0.93	0.91	0.92	0.85	0.92	0.90
ARS	0.83	0.97	0.95	0.92	0.94	0.93	0.96	0.98	0.93	0.93
Mean	0.83	0.92	0.93	0.90	0.93	0.92	0.94	0.91	0.93	
			Systems of cultivation	Nutrient management options	Interaction					
		SEM	0.01	0.02	0.03					
		CD (*P* = 0.05)	0.03	0.05	0.08					
Hull yield during 2014 (Mg ha^−1^)										
PTR	0.79	0.89	0.95	0.88	0.92	0.89	0.93	0.90	0.95	0.90
SRI	0.81	0.86	0.91	0.92	0.92	0.89	0.92	0.84	0.92	0.89
ARS	0.80	0.95	0.95	0.93	0.93	0.91	0.95	0.96	0.93	0.92
Mean	0.80	0.90	0.94	0.91	0.92	0.90	0.93	0.90	0.93	
			Systems of cultivation	Nutrient management options	Interaction					
		SEM	0.01	0.01	0.02					
		CD (*P* = 0.05)	0.03	0.04	0.06					

^a)^ PTR, puddled transplanted rice; SRI, system of rice intensification; ARS, aerobic rice system

^b)^ Recommended dose of nutrients (120 kg nitrogen ha^−1^ and 25.8 kg phosphorus ha^−1^)

^c)^ 5 kg Zn ha^−1^ through ZnSO_4_·7H_2_O

^d)^ *Anabaena* sp. (CR1) + *Providencia* sp. (PR3) consortium

^e)^ *Anabaena*–*Pseudomonas* biofilm formulation.

### 2.2 Water Productivity of Rough Rice

**Figure 1:**
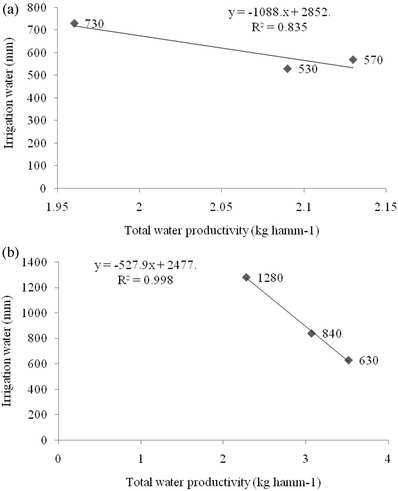
Relationship between irrigation water used and total water productivity in a) 2013 and b) 2014.

**Figure 2:**
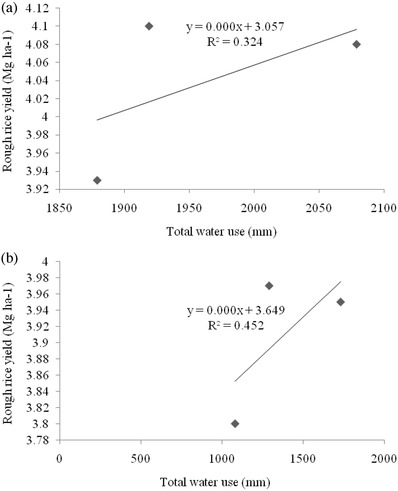
Relationship between rough rice yield and total water used in rice production during a) 2013 and b) 2014.

The highest total water productivity was recorded in the SRI method (2.13 kg ha^−1^ mm^−1^), which was statistically superior to ARS (2.09 kg ha^−1^ mm^−1^) (**Table** **6**), and both these methods recorded significantly higher total water productivity over PTR (1.96 kg ha^−1^ mm^−1^). The treatment containing RDN + Zn in SRI produced higher rough rice per unit of water consumed (2.27 kg ha^−1^ mm^−1^), which was closely followed by 75% RDN + An–Ps biofilm formulation + Zn and 75% RDN + CR1 + PR3 + Zn in SRI during the first year. During the second year, RDN + Zn in ARS performed better with highest total water productivity (3.79 kg ha^−1^ mm^−1^) and remained on par with 75% RDN + An–Ps biofilm formulation + Zn and 75% RDN + CR1 + PR3 + Zn. These three treatments were superior to all the other treatments during both the years in all CEMs. The total water productivity of rough rice was negatively correlated with irrigation water used during both the years with stronger negative correlation in the second year (*R* ^2^ = 0.99) than in the first year (*R* ^2^ = 0.83) (**Figure** **1**). The rough rice yield positively correlated with total water used during both the years (**Figure** **2**). The irrigation water productivity was higher than the total water productivity in all the three CEMs, in both the years of study (**Table** **7**). During the first year, irrigation water productivity was higher by 3.63 kg ha^−1^ mm^−1^ in PTR, 5.05 kg ha^−1^ mm^−1^ in SRI, and 5.32 kg ha^−1^ mm^−1^ in ARS than total water productivity, while during the second year it was higher by 0.81, 1.66, and 2.52 kg ha^−1^ mm^−1^ in PTR, SRI, and ARS, respectively. The irrigation water productivity of RDN + Zn was higher by 4.97 kg ha^−1^ mm^−1^ over total water productivity, while it was higher by 4.94 and 4.95 kg ha^−1^ mm^−1^ in 75% RDN + An–Ps biofilm formulation + Zn and 75% RDN + CR1 + PR3 + Zn, respectively, during the first year. The increase in irrigation water productivity over total water productivity during the second year was 1.79, 1.77, and 1.77 kg ha^−1^ mm^−1^, respectively, for the above‐mentioned treatments; these treatments recorded significantly higher irrigation water productivity than rest of the treatments in all CEMs in both the years of study.

**Table 6:** Influence of crop establishment methods and nutrient management options on total water productivity of rough rice (kg ha^−1^ mm^−1^) in 2013 and 2014

Treatment^a)^	Control (N_0_P_0_Zn_0_)	100% RDN	100% RDN^b)^ + Zn^c)^	75% RDN	75% RDN + Zn	75% RDN + CR1 + PR3^d)^	75% RDN + CR1 + PR3^d)^ + Zn	75% RDN + An–Ps^e)^ biofilm formulation	75% RDN + An–Ps^e)^ biofilm formulation + Zn	Mean
Total water productivity of rough rice (kg ha^−1^ mm^−1^) in 2013										
PTR	1.55	2.03	2.09	1.86	1.94	2.01	2.08	2.01	2.08	1.96
SRI	1.77	2.19	2.27	2.01	2.10	2.17	2.25	2.17	2.26	2.13
ARS	1.71	2.16	2.22	1.97	2.07	2.13	2.21	2.13	2.21	2.09
Mean	1.68	2.12	2.19	1.95	2.04	2.10	2.18	2.10	2.18	
			Systems of cultivation	Nutrient management options	Interaction					
		SEM	0.01	0.01	0.03					
		CD (*P* = 0.05)	0.03	0.04	0.07					
Total water productivity of rough rice (kg ha^−1^ mm^−1^) in 2014										
PTR	1.75	2.33	2.46	2.20	2.24	2.31	2.44	2.32	2.43	2.28
SRI	2.49	3.13	3.31	2.95	3.01	3.10	3.27	3.11	3.28	3.07
ARS	2.80	3.61	3.79	3.38	3.47	3.54	3.75	3.57	3.74	3.52
Mean	2.35	3.02	3.19	2.84	2.90	2.98	3.15	3.00	3.15	
			Systems of cultivation	Nutrient management options	Interaction					
		SEM	0.01	0.01	0.02					
		CD (*P* = 0.05)	0.03	0.04	0.06					

^a)^ PTR, puddled transplanted rice; SRI, system of rice intensification; ARS, aerobic rice system

^b)^ Recommended dose of nutrients (120 kg nitrogen ha^−1^ and 25.8 kg phosphorus ha^−1^)

^c)^ 5 kg Zn ha^−1^ through ZnSO_4_·7H_2_O

^d)^ *Anabaena* sp. (CR1) + *Providencia* sp. (PR3) consortium

^e)^ *Anabaena–Pseudomonas* biofilm formulation.

**Table 7:** Influence of crop establishment methods and nutrient management options on irrigation water productivity of rough rice (kg ha^−1^ mm^−1^) in 2013 and 2014

Treatment^a)^	Control (N_0_P_0_Zn_0_)	100% RDN	100% RDN^b)^ + Zn^c)^	75% RDN	75% RDN + Zn	75% RDN + CR1 + PR3^d)^	75% RDN + CR1 + PR3^d)^ + Zn	75% RDN + An–Ps^e)^ biofilm formulation	75% RDN + An–Ps^e)^ biofilm formulation + Zn	Mean
Irrigation water productivity of rough rice (kg ha^−1^ mm^−1^) in 2013										
PTR	4.42	5.77	5.97	5.31	5.52	5.72	5.94	5.73	5.93	5.59
SRI	5.96	7.37	7.64	6.78	7.07	7.32	7.58	7.31	7.63	7.18
ARS	6.06	7.65	7.88	7.00	7.35	7.54	7.85	7.55	7.84	7.41
Mean	5.48	6.93	7.16	6.36	6.65	6.86	7.12	6.86	7.13	
			Systems of cultivation	Nutrient management options	Interaction					
		SEM	0.02	0.04	0.07					
		CD (*P* = 0.05)	0.09	0.12	0.21					
Irrigation water productivity of rough rice (kg ha^−1^ mm^−1^) in 2014										
PTR	2.38	3.17	3.34	2.98	3.04	3.14	3.31	3.15	3.30	3.09
SRI	3.83	4.81	5.09	4.54	4.63	4.77	5.02	4.78	5.05	4.73
ARS	4.81	6.19	6.51	5.80	5.95	6.08	6.44	6.12	6.43	6.04
Mean	3.68	4.72	4.98	4.44	4.54	4.66	4.92	4.69	4.92	
			Systems of cultivation	Nutrient management options	Interaction					
		SEM	0.01	0.02	0.03					
		CD (*P* = 0.05)	0.04	0.05	0.09					

^a)^ PTR, puddled transplanted rice; SRI, system of rice intensification; ARS, aerobic rice system

^b)^ Recommended dose of nutrients (120 kg nitrogen ha^−1^ and 25.8 kg phosphorus ha^−1^)

^c)^ 5 kg Zn ha^−1^ through ZnSO_4_·7H_2_O

^d)^ *Anabaena* sp. (CR1) + *Providencia* sp. (PR3) consortium

^e)^ *Anabaena*–*Pseudomonas* biofilm formulation.

In this study, both Zn fertilization and microbial inoculation save substantial quantities of water (Tables 6 and 7). The highest increase in total and irrigation water productivity due to Zn fertilization was recorded when Zn was applied with 75% RDN (0.10 kg ha^−1^ mm^−1^) in ARS in the first year and 75% RDN + CR1 + PR3 (0.21 kg ha^−1^ mm^−1^) in ARS during the second year. This leads to savings of 245 and 158 L of total water kg^−1^ of rough rice produced in the first and second years, respectively. Similarly, the application of MI1 saved 381–401 and 102–135 L of total and irrigation water kg^−1^ of rough rice produced in the first year, while savings in the second year were 134–216 and 79–171 L kg^−1^ of rough rice produced.

### 2.3 Water Productivity of Brown Rice

The production of brown rice required greater amounts of water to produce 1 kg of rice than rough rice. The SRI method (1.67 kg ha^−1^ mm^−1^) gave significantly higher total water productivity in the first year, while ARS was found to be superior in the second year, with total water productivity of 2.66 kg ha^−1^ mm^−1^ (**Table** **8**). Among the nutrient management options, total water productivity was found highest in RDN + Zn in the SRI method (1.80 kg ha^−1^ mm^−1^) in the first year and in the ARS method (1.91 kg ha^−1^ mm^−1^) during the second year. This treatment remained statistically on par with that of 75% RDN + An–Ps biofilm formulation + Zn and 75% RDN + CR1 + PR3 + Zn in all CEMs. These three treatments recorded significantly higher total water productivity than all other treatments in all CEMs. With regard to the irrigation water productivity, ARS performed better during both the years with higher water productivity of 5.65 and 4.57 kg ha^−1^ mm^−1^ during the first and second years, respectively, which can be mainly due to lower amounts of irrigation water required in ARS in both the years, than PTR and SRI (**Table** **9**). In case of nutrient management treatments, the trend was found to be similar to that of total water productivity with highest irrigation water productivity in RDN + Zn applied in ARS (6.09 and 5.00 kg ha^−1^ mm^−1^) during both the years.

**Table 8:** Influence of crop establishment methods and nutrient management options on total water productivity of brown rice (kg ha^−1^ mm^−1^) in 2013 and 2014

Treatment^a)^	Control (N_0_P_0_Zn_0_)	100% RDN	100% RDN^b)^ + Zn^c)^	75% RDN	75% RDN + Zn	75% RDN + CR1 + PR3^d)^	75% RDN + CR1 + PR3^d)^ + Zn	75% RDN + An–Ps^e)^ biofilm formulation	75% RDN + An–Ps^e)^ biofilm formulation + Zn	Mean
Total water productivity of brown rice (kg ha^−1^ mm^−1^) in 2013										
PTR	1.16	1.59	1.64	1.45	1.49	1.57	1.64	1.57	1.63	1.53
SRI	1.34	1.73	1.80	1.54	1.62	1.70	1.77	1.72	1.79	1.67
ARS	1.27	1.64	1.72	1.48	1.57	1.63	1.70	1.61	1.72	1.59
Mean	1.25	1.65	1.72	1.49	1.56	1.63	1.70	1.63	1.71	
			Systems of cultivation	Nutrient management options	Interaction					
		SEM	0.01	0.01	0.02					
		CD (*P* = 0.05)	0.02	0.03	0.05					
Total water productivity of brown rice (kg ha^−1^ mm^−1^) in 2014										
PTR	1.30	1.82	1.92	1.69	1.71	1.79	1.90	1.81	1.88	1.76
SRI	1.87	2.46	2.61	2.24	2.30	2.41	2.55	2.46	2.57	2.39
ARS	2.06	2.73	2.91	2.51	2.61	2.70	2.87	2.68	2.88	2.66
Mean	1.74	2.34	2.48	2.15	2.20	2.30	2.44	2.32	2.44	
			Systems of cultivation	Nutrient management options	Interaction					
		SEM	0.01	0.01	0.02					
		CD (*P* = 0.05)	0.02	0.03	0.06					

^a)^ PTR, puddled transplanted rice; SRI, system of rice intensification; ARS, aerobic rice system

^b)^ Recommended dose of nutrients (120 kg nitrogen ha^−1^ and 25.8 kg phosphorus ha^−1^)

^c)^ 5 kg Zn ha^−1^ through ZnSO_4_·7H_2_O

^d)^ *Anabaena* sp. (CR1) + *Providencia* sp. (PR3) consortium

^e)^ *Anabaena–Pseudomonas* biofilm formulation.

**Table 9:** Influence of crop establishment methods and nutrient management options on irrigation water productivity of brown rice (kg ha^−1^ mm^−1^) in 2013 and 2014

Treatment^a)^	Control (N_0_P_0_Zn_0_)	100% RDN	100% RDN^b)^ + Zn^c)^	75% RDN	75% RDN + Zn	75% RDN + CR1 + PR3^d)^	75% RDN + CR1 + PR3^d)^ + Zn	75% RDN + An–Ps^e)^ biofilm formulation	75% RDN + An–Ps^e)^ biofilm formulation + Zn	Mean
Irrigation water productivity of brown rice (kg ha^−1^ mm^−1^) in 2013										
PTR	3.29	4.53	4.68	4.12	4.25	4.48	4.66	4.47	4.63	4.35
SRI	4.50	5.83	6.05	5.19	5.45	5.72	5.97	5.81	6.01	5.61
ARS	4.49	5.82	6.09	5.26	5.57	5.79	6.04	5.71	6.09	5.65
Mean	4.09	5.39	5.61	4.86	5.09	5.33	5.56	5.33	5.58	
			Systems of cultivation	Nutrient management options	Interaction					
		SEM	0.02	0.03	0.05					
		CD (*P* = 0.05)	0.07	0.09	0.15					
Irrigation water productivity of brown rice (kg ha^−1^ mm^−1^) in 2014										
PTR	1.77	2.48	2.61	2.29	2.32	2.44	2.58	2.45	2.56	2.39
SRI	2.87	3.79	4.01	3.44	3.54	3.71	3.93	3.79	3.95	3.67
ARS	3.55	4.69	5.00	4.32	4.48	4.63	4.93	4.61	4.95	4.57
Mean	2.73	3.65	3.87	3.35	3.44	3.59	3.81	3.62	3.82	
			Systems of cultivation	Nutrient management options	Interaction					
		SEM	0.01	0.02	0.03					
		CD (*P* = 0.05)	0.04	0.05	0.08					

^a)^ PTR, puddled transplanted rice; SRI, system of rice intensification; ARS, aerobic rice system

^b)^ Recommended dose of nutrients (120 kg nitrogen ha^−1^ and 25.8 kg phosphorus ha^−1^)

^c)^ 5 kg Zn ha^−1^ through ZnSO_4_·7H_2_O

^d)^ *Anabaena* sp. (CR1) + *Providencia* sp. (PR3) consortium

^e)^ *Anabaena–Pseudomonas* biofilm formulation.

### 2.4 Water Saving due to CEMs

In the first year of the experiment, SRI saved 21.9% irrigation water, while ARS saved 27.4% irrigation water over PTR (**Table** **10**), while during the second year, SRI saved 37.4% irrigation water and ARS saved 50.8% irrigation water over PTR. The total water savings in both SRI and ARS were very low during the first year (7.7% and 7.9%, respectively). In the second year, savings of total water were 25.4% and 37.5% in SRI and ARS, respectively, over PTR.

**Table 10:** Effect of rice cultivation system on saving of water in rice during the first and second years of experiment

Rice cultivation system^a)^	Irrigation water used [mm]	Rainfall during crop growing season [mm]	Total water used [mm]	Irrigation water saving [%]	Total water saving [%]
2013					
PTR	730	1349	2079	–	–
SRI	570	1349	1919	21.9	7.7
ARS	530	1349	1879	27.4	7.9
2014					
PTR	1280	451	1731	–	–
SRI	840	451	1291	37.4	25.4
ARS	630	451	1081	50.8	37.5

^a)^ PTR, puddled transplanted rice; SRI, system of rice intensification; ARS, aerobic rice system.

### 2.5 Agronomic Use Efficiency of Nitrogen and Phosphorus

The agronomic use efficiency of nitrogen (AUEN) was higher during the second year (**Table** **11**) and CEMs were not able to influence AUEN significantly. Among the nutrient management options, 75% RDN + An–Ps biofilm formulation both with and without Zn recorded significantly higher AUEN, as compared to other treatments. The interaction between CEMs and nutrient management options on AUEN was found significant in both the years. On comparing PTR with SRI, all the treatments were found significantly higher in PTR, except two treatments, namely, RDN with and without Zn in the first year, while during the second year, all the treatments were statistically superior in PTR, than the SRI system. Based on the performance of treatments in ARS and SRI, all the treatments were statistically at par in both the years. However, all the treatments in PTR exhibited superior performance, as compared to those applied in ARS during the second year, while during the first year, three treatments, namely, RDN with and without Zn and 75% RDN + Zn, remained at par in both the systems, with significantly higher AUEN in PTR.

**Table 11:** Influence of cultivation systems and nutrient management options on agronomic use efficiency of nitrogen in rice during 2013 and 2014

Treatment^a)^	Control (N_0_P_0_Zn_0_)	RDN^b)^	RDN + Zn^c)^	75% RDN	75% RDN + Zn	75% RDN + CR1 + PR3^d)^	75% RDN + CR1 + PR3^d)^ + Zn	75% RDN + An–Ps^e)^ biofilm formulation	75% RDN + An–Ps^e)^ biofilm formulation + Zn	Mean
2013										
PTR	0.00	8.22	9.42	7.19	8.95	10.59	12.33	10.60	12.23	8.84
SRI	0.00	6.67	7.94	5.19	7.02	8.59	10.22	8.49	10.53	7.18
ARS	0.00	7.03	8.03	5.52	7.58	8.70	10.52	8.79	10.45	7.40
Mean	0.00	7.31	8.46	5.97	7.85	9.30	11.02	9.29	11.07	
			Systems of cultivation	Nutrient management options	Interaction					
		SEM	1.404	0.324	0.5609					
		CD (*P* = 0.05)	5.513	0.921	1.5948					
2014										
PTR	0.00	8.40	10.27	8.53	9.30	10.72	13.17	10.95	13.03	9.38
SRI	0.00	6.88	8.83	6.57	7.41	8.76	11.10	8.88	11.36	7.75
ARS	0.00	7.24	8.91	6.90	7.97	8.87	11.40	9.17	11.29	7.97
Mean	0.00	7.50	9.34	7.33	8.23	9.45	11.89	9.67	11.89	
			Systems of cultivation	Nutrient management options	Interaction					
		SEM	0.927	0.219	0.3798					
		CD (*P* = 0.05)	3.642	0.624	1.0800					

^a)^ PTR, puddled transplanted rice; SRI, system of rice intensification; ARS, aerobic rice system

^b)^ Recommended dose of nutrients (120 kg nitrogen ha^−1^ and 25.8 kg phosphorus ha^−1^)

^c)^ 5 kg Zn ha^−1^ through ZnSO_4_·7H_2_O

^d)^ *Anabaena* sp. (CR1) + *Providencia* sp. (PR3) consortium

^e)^ *Anabaena*–*Pseudomonas* biofilm formulation.

The agronomic use efficiency of phosphorus (AUEP) was higher than AUEN in both the years, which was mainly due to the lower quantity of P applied, as compared to N. Among the 2 years, AUEP was higher in the second year, with a similar trend to that of AUEN. All the CEMs remained on par in both the years in AUEP (**Table** **12**). Among the nutrient management options, 75% RDN + An–Ps biofilm formulation with and without Zn recorded significantly higher AUEP than other options. The interaction effect between cultivation systems and nutrient management options was found to be significant in both the years. All the treatments performed better in PTR than SRI, except three treatments, namely, RDN with and without Zn and 75% RDN + An–Ps biofilm formulation + Zn in the first year, while during the second year, PTR was found to be superior to SRI. PTR was found to be superior to ARS in the second year, while statistically at par in the first year.

**Table 12:** Influence of cultivation systems and nutrient management options on agronomic use efficiency of phosphorus in rice during 2013 and 2014

Treatment^a)^	Control (N_0_P_0_Zn_0_)	RDN^b)^	RDN + Zn^c)^	75% RDN	75% RDN + Zn	75% RDN + CR1 + PR3^d)^	75% RDN + CR1 + PR3^d)^ + Zn	75% RDN + An–Ps^e)^ biofilm formulation	75% RDN + An–Ps^e)^ biofilm formulation + Zn	Mean
2013										
PTR	0.00	38.24	43.80	33.44	41.62	49.27	57.36	49.30	56.88	41.10
SRI	0.00	31.01	36.95	24.13	32.66	39.97	47.55	39.48	48.96	33.41
ARS	0.00	15.45	37.34	25.68	35.25	40.48	48.92	40.86	48.61	32.51
Mean	0.00	28.23	39.36	27.75	36.51	43.24	51.28	43.22	51.48	
			Systems of cultivation	Nutrient management options	Interaction					
		SEM	6.563	1.788	3.0969					
		CD (*P* = 0.05)	25.768	5.084	8.8059					
2014										
PTR	0.00	39.08	47.78	39.67	43.26	49.87	61.27	50.94	60.59	43.61
SRI	0.00	31.98	41.06	30.54	34.47	40.74	51.63	41.29	52.83	36.06
ARS	0.00	33.66	41.45	32.09	37.05	41.26	53.01	42.67	52.49	37.07
Mean	0.00	34.91	43.43	34.10	38.26	43.96	55.30	44.97	55.30	
			Systems of cultivation	Nutrient management options	Interaction					
		SEM	4.314	1.020	1.7667					
		CD (*P* = 0.05)	16.937	2.900	5.0235					

^a)^ PTR, puddled transplanted rice; SRI, system of rice intensification; ARS, aerobic rice system

^b)^ Recommended dose of nutrients (120 kg nitrogen ha^−1^ and 25.8 kg phosphorus ha^−1^)

^c)^ 5 kg Zn ha^−1^ through ZnSO_4_·7H_2_O

^d)^ *Anabaena* sp. (CR1) + *Providencia* sp. (PR3) consortium

^e)^ *Anabaena*–*Pseudomonas* biofilm formulation.

## 3 Discussion

### 3.1 Rice Yield

In this investigation, the variation observed in the yield of the 2 years can be attributed to the weather variation, mainly in terms of rainfall. However, an attempt was made to maintain a favorable environment during the second year by providing irrigation water, which was responsible for reducing the difference in yield between the first and second years in this study. The effect of rainfall on rice yield is well documented.^30^, ^31^, ^32^ Over a 10‐year study, Sankar Maruthi et al.^33^ also found an increasing trend in rice yield with higher rainfall at three different locations (Varanasi, Faizabad, and Ranchi) in India. Among CEMs, the superiority of SRI, over ARS, in yielding capacity was also reported by Singh^34^ and Geethalakshmi et al.^35^ Singh^34^ also found that SRI and PTR systems remained on par with respect to rough rice yield. The superiority of PTR system over ARS in terms of rough rice yield was reported by Ram et al.^36^ A significant response of rice to Zn application was obtained in all the treatments over no Zn application (Table 1). Yield improvement due to zinc application was reported by Shivay et al.^37^ and Shivay and Prasad.^38^ The rough and brown rice yields in the microbial inoculation applied treatments were similar to that obtained with the application of RDN. Improvement in rice yields due to microbial inoculation of CR2, CR1 (both *Anabaena* spp.), and PR10 (*Ochrobactrum* sp.) with two‐thirds of nitrogenous dose was earlier reported by Prasanna et al.^25^ Variations in hulling and milling percentage among CEMs were responsible for the difference in brown and milled rice yield between them. Higher milling recovery in PTR and SRI over ARS leads to higher milled rice yield. The effect of CEMs on hulling recovery of rice was also mentioned by Mahendra Kumar et al.^39^ Along with the CEMs, Zn fertilization and the use of microbial inoculations also had a significant impact on hulling and milling percentage, which was seen from the variation in yield of brown and milled rice. Higher hulling percentage due to zinc application^38^ and microbial inculcation^40^ has been reported. Hull yield was not affected significantly by the cultivation systems although the highest hull yield in ARS can be attributed to the lower hulling recovery than PTR and SRI.

### 3.2 Water Productivity of Rough and Brown Rice

The variation in water productivity among CEMs was mainly due to the differences in the yield and water used in the production process. There was higher water productivity in the SRI method over PTR, although both recorded nearly equal yield, but lower water use in SRI than PTR. The higher water productivity in ARS can be solely due to lower amounts of water used to produce 1 kg rice in ARS than PTR. Higher water productivity in SRI was observed by Thakur et al.^41^ and Zhao et al.^42^ also reported higher irrigation and total water use efficiency in SRI over PTR. The superiority of ARS in improving water productivity over PTR was discussed by Singh.^34^ Total water productivity was higher in the second year, which can be attributed to lower amount of total water used. The higher water productivity in treatment involving application of Zn and microbial inoculation showed a positive correlation with increase in rough rice yield by both Zn and microbial inoculation, illustrating the benefits with reduced resources. The irrigation water productivity was higher than total water productivity due to the lower quantity of irrigation water than total water used in the production of same quantity of rice. Suryavanshi et al.^43^ and Singh^34^ also reported higher irrigation water productivity than total water productivity in their study. The better correlation of irrigation water with total water productivity in the second year was due to the major share of irrigation water to the total water supply to rice, as rainfall was lower in the second year (Figure 1). At the same time, a significant positive correlation of rough rice with total water used in the second year (*R* ^2^ = 0.45) than the first year (*R* ^2^ = 0.32) could also be due to the greater contribution of irrigation water to the total water supply to rice in the second year (Figure 1).

A higher water requirement for the production of 1 kg brown rice was mainly due to lower yield of brown rice than rough rice, while water consumption was similar in both cases. The total water productivity of brown rice in SRI was less by 0.46 kg ha^−1^ mm^−1^ as compared to rough rice. Similarly, in ARS and PTR, the total water productivity of brown rice was less by 0.50 and 0.43 kg ha^−1^ mm^−1^ than for rough rice. The superiority of SRI in total water productivity of brown rice in the first year was due to lower water used than PTR and higher yield over ARS, while superiority of ARS in the second year over PTR and SRI was on account of lower water used in ARS than both SRI and PTR. Along with CEMs, the positive effect of Zn fertilization and microbial inoculation on the yield of rough rice and milling percentage can be major factors contributing to the increase in total water productivity of brown rice. The improvement in hulling recovery of rice by application of Zn may be responsible for higher brown rice yield, as reported earlier.^44^

### 3.3 Water Saving and Nutrient Use Efficiency

In terms of water savings due to CEMs, the lower depth of irrigation water application in SRI than PTR during the initial growth period was responsible for the saving of water in SRI. In ARS, the absence of nursery, no puddling, and maintaining arable soil lead to saving of more water than both PTR and SRI. In the second year, water saving was higher than the first year; this was mainly due to lower share of rainfall and maximum share of irrigation water in total water used in the production of rice. Singh^34^ reported water savings in SRI and ARS over PTR in both the years, in their 2‐year study at New Delhi. Saving of water in SRI over PTR has also been documented.^42^, ^45^, ^46^ Among the treatments of Zn fertilization and microbial inoculations, yield improvement was more in microbial inoculation, which translated into greater water savings. The quantity of total water saving is less and irrigation water saving is more during the second year than the first year; this is on account of the lower rainfall received during the second year.

The variation in AUEN years was mainly due to greater variation in the yield of treated plots and control plot, as a result of unfavorable weather conditions. The superiority of 75% RDN + An–Ps biofilm formulation in AUEN and AUEP is the result of lower N and P application rate than RDN, but statistically at par yields with RDN. A higher AUEN at a low rate of N application was also reported by Zhao et al.^42^ and Shivay et al.^47^; variation in AUEN among CEMs was also reported by Ali et al.^48^ and Satyanarayana et al.^49^ It is well documented that these microbial inoculants, which include nitrogen fixers, can lead to a saving of 25% N, without negative effects on yields.^25^, ^26^, ^27^, ^28^, ^29^ Additionally, the biofilm inoculants have the potential to provide fixed nitrogen in a sustained manner to the crop, up to the harvest stage.^28^

## 4 Conclusions

From our study, it can be concluded that changing the CEM from PTR to SRI increases water productivity without any reduction in yield, while ARS increases water productivity with yield penalty. The highest total water productivity was observed with RDN + Zn in SRI methods in the first year, while in the second year, the same treatment applied in ARS recorded highest total water productivity of both rough and brown rice. In case of irrigation water productivity of rough and brown rice, RDN + Zn was found to be superior in ARS. Application of zinc and microbial inoculation increases the yield of rough rice and also has a positive effect on increasing the hulling and milling percentage of rice, leading to higher brown and milled rice yields. This increase in yield of rough, brown, and milled rice due to Zn application and use of microbial inoculation can also reflect the indirect effect of improved total and irrigation water productivity, leading to economy in water use. An increase in the agronomic use efficiency of N and P in microbial inoculation treatments over RDN makes them effective and profitable inputs for saving both water and nutrients.

## 5 Experimental Section

*Experimental Site and Meteorological Condition*: The experiment was conducted on the research farm of the ICAR—Indian Agricultural Research Institute, New Delhi, India. This farm was situated at a latitude of 28°38′ N and longitude of 77°10′ E, and altitude of 228.6 m above the mean sea level (Arabian Sea). The experiment was carried out during the rainy (wet) season (June to October) for two consecutive years (2013 and 2014) in the same field. Before the start of the experiment, the soil was analyzed for its important physical and chemical properties (0–15 cm depth). The texture of soil (Typic Ustochrept) was sandy clay loam with a mechanical composition of 51.4% sand, 22.2% silt, and 26.4% clay with moderate water holding capacity and laser leveled topography. The soils of the experimental field (0–15 cm depth) had 0.60% organic C,^50^ 257 kg ha^−1^ alkaline permanganate oxidizable N,^51^17 kg ha^−1^ available P (Olsen's method),^52^ 327 kg ha^−1^ 1 n ammonium acetate exchangeable K,^53^ and 0.85 mg kg^−1^ of available zinc.^54^ The pH of the soil was 7.6 (1:2.5 soil and water ratio).

The climate of Delhi is of subtropical and semiarid type with hot and dry summer and cold winter and is categorized under the agroclimatic zone “Trans‐Gangetic Plains.” The mean annual normal rainfall is 650 mm, with August being the wettest month. The annual mean pan evaporation is about 850 mm. Meteorological data were recorded in the observatory of ICAR—Indian Agricultural Research Institute, during the crop growing period. In the first year of experiment, the total rainfall received was 1349.8 mm with 39 rainy days, while during the second year, 451.8 mm of rainfall was received with 22 rainy days.

*Experimental Details*: A field experiment was planned in a split‐plot design involving three CEMs as the main plots, namely, PTR, SRI, and ARS. In each of these CEMs, nine nutrient management treatments were applied and all the treatments were taken in triplicate. Subplot nutrient management treatments included T1, control—no fertilizer (N_0_P_0_Zn_0_); T2, RDN (120 kg N ha^−1^ and 25.8 kg P ha^−1^); T3, T2 + zinc (5 kg ha^−1^); T4, 75% RDN; T5, T4 + zinc (5 kg ha^−1^); T6, 75% RDN + CR1 + PR3; T7, T6 + zinc (5 kg ha^−1^); T8, 75% RDN + An–Ps biofilm formulation; and T9, T8 + zinc (5 kg ha^−1^). Potassium was applied uniformly in all treatments at the rate of 49.8 kg K ha^−1^. The details of the preparation of these inoculants and their formulations are given in the previous investigations.^26^, ^27^, ^28^, ^29^ Both these formulations were prepared by mixing with vermiculite (hydrous phyllosilicate mineral):compost (1:1) as the carrier. The paddy straw compost was C/N 16.22 and humus 13.8% (pH 7.34), and the cyanobacterial, fungal, and bacterial colony forming units in the formulations were 10^4^, 10^5^, and 10^8^ g^−1^ carrier, respectively, as optimized in earlier studies.^28^, ^29^ All the bacterial/cyanobacterial strains were maintained in the culture collection of the Division of Microbiology, ICAR—Indian Agricultural Research Institute, New Delhi, India. These consortia/strains/biofilms have been used in several experiments earlier and proven to be plant growth promoting biofertilizing options.^25^, ^26^, ^27^, ^28^, ^29^

Details of the seed and sowing/transplanting specifications are given in **Table** **13**. In this experiment, the scented rice variety “Pusa Sugandh 5”^12^, ^55^ was planted during both the years. To maintain the same growth stage of rice in all the three CEMs, sowing of rice in ARS in main field and sowing of rice in the nursery for transplanting in PTR and SRI were done on the same date (the third week of June in both the years of study). The main field of ARS was thoroughly prepared by plowing once and harrowing twice along with planking. For the application of microbial inoculants, presoaked seeds were treated with thick slurry of microbial cultures, using 1% carboxylmethyl cellulose (CMC) as a sticker. Seeds were manually drilled in plots at a row spacing of 20 cm and seeding rate was 60 kg ha^−1^. Field was kept under aerobic condition throughout the whole growing season and water was applied (2 cm) as and when soil moisture depletion reached 50% of the field capacity; the field was drained, whenever heavy rains occurred. The depth of water application was higher (5 cm) during the panicle emergence to completion of grain filling stage. Irrigation was withheld 10–12 d ahead of harvesting.

**Table 13:** Seed and sowing specification of rice in three different systems of cultivation

System of cultivation^a)^	Method of sowing	Seed rate [kg ha^−1^]	Spacing	Age of seedling	Seedling per hill	Water management
PTR	Transplanting	20	20 × 15	23–25 d old	2–3	5 cm puddled and saturated
SRI	Transplanting	5	20 × 20	13–14 d old	1	2 cm up to panicle initiation and 5 cm thereafter
ARS	Direct sowing of seed in main field	60	20 cm row to row	Direct sowing of seed in field	–	2 cm to maintain field capacity moisture level, nonsaturated, and nonpuddled

^a)^ PTR, puddled transplanted rice; SRI, system of rice intensification; ARS, aerobic rice system.

In the SRI method, 13–14‐d‐old seedlings were transplanted at 20 cm × 20 cm spacing in puddled soil and a saturated field condition was maintained from transplanting to panicle initiation stage by applying 2 cm depth of water when field soil developed fine cracks. After panicle initiation, 5 cm water was applied as and when field soil developed fine cracks. Irrigation was withheld 10–12 d before harvesting. Under PTR, 23–25‐d‐old seedlings were transplanted in puddled soil at 20 cm × 15 cm spacing and a water depth of 5 cm was applied at each irrigation. Irrigation was stopped 10–12 d before harvesting. For the application of microbial inoculants, a thick slurry of microbial cultures was made by using water along with 1% CMC as a sticker. The rice seedlings in PTR and SRI treatments were dipped for half an hour before transplanting. The total number of irrigations and amount of water applied varied with CEMs and rainfall during growing season. In PTR, 11 and 18 irrigations were applied in first and second years, while in SRI, 11 and 20 irrigations, and in ARS, 10 and 24 irrigations were applied in the first and second years of the study, respectively. Two hand weedings in PTR and SRI and three hand weedings in ARS were done. Net plot size of each subplot was 8 m^2^.

*Observations*: The crop was manually harvested with sickles at ripening stage. The plants from the border rows were harvested first and removed from field. Thereafter, the net plot area was harvested and produce was left in the field, until it dried. Threshing was done manually and the rough rice yield was recorded after cleaning, at 14% moisture, and expressed in terms of Mg ha^−1^. For the calculation of yield of brown rice, milled rice, hull, and bran from rough rice, hulling and milling of rice was done. For calculation of hulling percentage, sun‐dried samples of paddy, weighing 1000 g each from all the plots, were hulled in a mini “Satake Rice Mill”^56^ and hulling percentage was calculated as

(1) $$\text{hulling}\;\text{percentage}\;\left(\%\right)=\frac{\text{weight}\;\text{of}\;\text{brown}\;\text{rice}\;\left(\text{g}\right)}{\text{weight}\;\text{of}\;\text{rough}\;\text{rice}\;\left(\text{g}\right)}\;\times \;100$$

From the hulling percentage obtained, brown rice yield and yield of hull were calculated. For the calculations of milled rice yield and bran yield, the hulled brown rice was passed through “Satake Rice Whitening and Caking Machine”^56^ for 2 min. The polished rice was weighed and milling percentage was worked out by using the following formula. From milling, yield of white rice and bran was calculated

(2) $$\text{milling}\;\text{percentage}\;\left(\%\right)=\frac{\text{weight}\;\text{of}\;\text{milled}\;\text{rice}\;\left(\text{g}\right)}{\text{weight}\;\text{of}\;\text{rough}\;\text{rice}\;\left(\text{g}\right)}\;\times \;100$$

For the measurement of water productivity, water intakes from rainfall and irrigation water were measured. For the calculation of irrigation water intake, total number of irrigations and depth of water applied at each irrigation were measured using a meter scale, which had millimeter and centimeter marks. In each plot, the depth of water was measured at ten selected spots after irrigation, and on the basis of these observations, the mean depth of irrigation water was calculated for each plot. The other measurements were calculated as

(3) $$\begin{array}{l} \text{irrigation}\;\text{water}\;\text{used }\left(\text{mm}\right)\;=\;\text{total}\;\text{number}\;\text{of}\;\text{irrigations}\;\text{applied}\\ \times \;\text{average}\;\text{depth}\;\text{of}\;\text{irrigation}\;\text{applied}\;\\ \;\;\;\text{at}\;\text{each}\;\text{irrigation} \end{array}$$

(4) $$\begin{array}{l} \text{total}\;\text{water}\;\text{intake}\;\left(\text{mm}\right)\;=\;\text{total}\;\text{rainfall}\;\text{received}\;\text{during}\;\text{the}\;\text{crop}\;\text{growing}\;\text{period}\;\left(\text{mm}\right)\;\\ +\;\text{irrigation}\;\text{water}\;\text{used}\;\text{to}\;\text{raise}\;\text{the}\;\text{crop}\;\left(\text{mm}\right) \end{array}$$

(5) $$\text{total}\;\text{water}\;\text{productivity}\;\left(\text{kg}\;{\text{ha}}^{-1}\;{\text{mm}}^{-1}\right)=\frac{\text{grain}\;\text{yield}\;\left(\text{kg}\;{\text{ha}}^{-1}\right)}{\text{total}\;\text{water}\;\text{intake}\;\left(\text{mm}\right)}$$

(6) $$\text{irrigation}\;\text{water}\;\text{productivity}\;\left(\text{kg}\;{\text{ha}}^{-1}\;{\text{mm}}^{-1}\right)=\frac{\text{grain}\;\text{yield}\;\left(\text{kg}\;{\text{ha}}^{-1}\right)}{\text{irrigation}\;\text{water}\;\text{used}\;\left(\text{mm}\right)}$$

(7) $$\begin{array}{l} \text{irrigation}\;\text{water}\;\text{saving}\;\text{in}\;\text{SRI}\;\text{or}\;\text{ARS}\;\left(\%\right)\\ =\frac{(\text{irrigation}\;\text{water}\;\text{used}\;\text{in}\;\text{PTR}-\text{irrigation}\;\text{water}\;\text{used}\;\text{in}\;\text{SRI}\;\text{or}\;\text{ARS)}}{\text{irrigation}\;\text{water}\;\text{used}\;\text{in}\;\text{PTR}}\;\times \;100 \end{array}$$

(8) $$\begin{array}{l} \text{total}\;\text{water}\;\text{saving}\;\text{in}\;\text{SRI}\;\text{or}\;\text{ARS}\;\left(\%\right)\\ =\frac{(\text{total}\;\text{water}\;\text{used}\;\text{in}\;\text{PTR}-\text{total}\;\text{water}\;\text{used}\;\text{in}\;\text{SRI}\;\text{or}\;\text{ARS)}}{\text{total}\;\text{water}\;\text{used}\;\text{in}\;\;\text{PTR}}\;\times \;100 \end{array}$$

The nitrogen and phosphorus use efficiencies were calculated by using the following formula

(9) $$\text{agronomic}\;\text{use}\;\text{efficiency}\;\left(\text{kg}\;\text{grain}\;\text{increased}\;{\text{kg}}^{-1}\;\text{N/P}\;\text{applied}\right)=\frac{\left(Y_{\text{f}}-Y_{\text{c}}\right)}{N_{\text{a}}}$$

where *Y*_f_ and *Y*_c_ refer to rice grain yield in fertilizer applied and absolute control plot, respectively. *N*_a_ refers to amount of nutrient (N or P) applied through fertilizer.

*Statistical Analysis*: All the data obtained from the experiment that was conducted using the split‐plot design were statistically analyzed in Microsoft Excel using the *F*‐test, following the procedures outlined by Gomez and Gomez.^57^ The least significant difference values (*P* = 0.05) were used to determine the significance among the treatment means.

## Conflict of Interest

The authors declare no conflict of interest.

## Supporting information

Supplementary

## References

[1] Anonymous , Agricultural Statistics at a Glance, Directorate of Economics & Statistics, Department of Agriculture, Cooperation & Farmers Welfare, Ministry of Agriculture & Farmers Welfare, New Delhi, India 2016.

[2] The Fertilizer Association of India (FAI) , Fertilizer Statistics, 58th ed., The Fertilizer Association of India, New Delhi 2013.

[3] V. V. Krishna , N. G. Byju , S. Tamizheniyan , in Radcliffe's IPM World Textbook (Eds: Radcliffe E. B., Hutchison W. D., Cancelado R. E.), University of Minnesota, St. Paul, MN 2002.

[4] R. Prasad , Y. S. Shivay , D. Kumar , J. Pandey , in Textbook of Field Crop Production, Vol. 1 (Ed: Prasad R.), ICAR Publications, New Delhi, India 2012, pp. 1–65.

[5] B. S. Chauhan , G. Mahajan , V. Sardana , J. Timsina , M. L. Jat , Adv. Agron. 2012, 117, 315.

[6] P. K. Sharma , L. Bhushan , J. K. Ladha , R. K. Naresh , R. K. Gupta , B. V. Balasubramanian , B. A. M. Bouman , in Water‐Wise Rice Production (Eds: Bouman B. A. M., Hengsdijk H., Hardy B., Bindraban P. S., Toung T. P., Ladha J. K.), International Rice Research Institute, Los Banõs, Philippines 2002, pp. 223–235.

[7] R. Barker , D. Dawe , T. P. Tuong , S. I. Bhuiyan , L. C. Guerra , in Assessment and Orientation towards the 21st Century, FAO, Rome 1999, pp. 96–109.

[8] S. K. Jalota , A. Sood , G. B. S. Chahal , B. U. Choudhury , Agric. Water Manage. 2006, 84, 137.

[9] G. S. Hira , J. Crop Improv. 2009, 23, 136.

[10] G. S. Hira , S. K. Jalota , V. K. Arora , Research Bulletin, Department of Soils, Punjab Agricultural University, Ludhiana 2004, pp. 22–30.

[11] R. Kumar , R. Gopal , M. L. Jat , R. K. Gupta , Training Manual, Maize for Freshers, Directorate of Maize Research, New Delhi, India 2010.

[12] R. Prasad , Adv. Agron. 2011, 111, 208.

[13] N. Uphoff , R. Randriamiharisoa , in Water‐Wise Rice Production (Eds: Bouman B. A. M., Hengsdijk H., Hardy B., Bindraban P. S., Toung T. P., Ladha J. K.) International Rice Research Institute, Los Banõs, Philippines 2003.

[14] R. K. Mall , A. Gupta , R. Singh , R. S. Singh , L. S. Rathore , Curr. Sci. 2006, 90, 1610.

[15] C. G. Madhusoodhanan , K. G. Sreeja , T. I. Eldho , Ambio 2016, 45, 725. 27170012 10.1007/s13280-016-0784-7 PMC5012999

[16] P. Kumar , P. K. Joshi , P. S. Brithal , Agric. Econ. Res. Rev. 2009, 22, 237.

[17] R. Bhattacharyya , B. N. Ghosh , P. K. Mishra , B. Mandal , C. S. Rao , D. Sarkar , K. Das , K. S. Anil , M. Lalitha , K. M. Hati , A. J. Franzluebbers , Sustainability 2015, 7, 3528.

[18] J. Timsina , D. J. Connor , Field Crops Res. 2001, 69, 93.

[19] N. K. Fageria , V. C. Baligar , Adv. Agron. 2005, 88, 97.

[20] D. Cordell , J. O. Drangert , S. White , Global Environ. Change 2009, 19, 292.

[21] J. Shen , L. Yuan , J. Zhang , H. Li , Z. Bai , X. Chen , W. Zhang , F. Zhang , Plant Physiol. 2011, 156, 997. 21571668 10.1104/pp.111.175232 PMC3135930

[22] M. G. Palmgren , S. Clemens , L. Williams , U. Kramer , S. Borg , K. Schjorring , D. Sanders , Trends Plant Sci. 2008, 13, 464. 18701340 10.1016/j.tplants.2008.06.005

[23] A. K. Shukla , S. K. Behera , Y. S. Shivay , P. Singh , A. K. Singh , Indian J. Agron. 2012, 57, 123.

[24] D. Kumar , Y. S. Shivay , S. Dhar , C. Kumar , R. Prasad , Proc. Natl. Acad. Sci. India, Sect. B: Biol. Sci. 2013, 83, 1.

[25] R. Prasanna , M. Joshi , A. Rana , Y. S. Shivay , L. Nain , World J. Microbiol. Biotechnol. 2012, 28, 1223. 22805842 10.1007/s11274-011-0926-9

[26] R. Prasanna , S. Pattnaik , T. C. K. Sugitha , L. Nain , A. K. Saxena , Folia Microbiol. 2011, 56, 49. 21448713 10.1007/s12223-011-0013-5

[27] L. Nain , A. Rana , M. Joshi , S. D. Jadhav , D. Kumar , Y. S. Shivay , S. Paul , R. Prasanna , Plant Soil 2010, 331, 217.

[28] R. Prasanna , A. Adak , S. Verma , N. Bidyarani , S. Babu , M. Pal , Y. S. Shivay , L. Nain , Ecol. Eng. 2015, 84, 532.

[29] A. Adak , R. Prasanna , S. Babu , N. Bidyarani , S. Verma , M. Pal , Y. S. Shivay , L. Nain , J. Plant Nutr. 2016, 39, 1216.

[30] K. Saito , B. Linquist , B. Keobualapha , K. Phanthaboon , T. Shiraiwa , T. Horie , Plant Soil 2006, 284, 175.

[31] H. Asada , J. Matsumoto , Clim. Res. 2009, 38, 249.

[32] M. Auffhammer , V. Ramanathan , J. R. Vincent , Clim. Change 2011, 111, 411.

[33] G. R. Sankar Maruthi , L. K. Sharma , S. K. Reddy , G. Pratibha , R. Shinde , S. R. Singh , A. K. Nema , R. P. Singh , B. S. Rath , A. Mishra , B. D. Behera , C. R. Subudhi , B. Singh , H. C. Singh , A. K. Singh , D. K. Rusia , M. S. Yadava , C. R. Thyagaraj , P. K. Mishra , M. Suma Chandrika , B. Venkateswarlu , Exp. Agric. 2013, 49, 161.

[34] Y. V. Singh , Paddy Water Environ. 2013, 11, 531.

[35] V. Geethalakshmi , T. Ramesh , A. Palamuthirsolai , Arch. Agron. Soil Sci. 2011, 57, 159.

[36] M. Ram , H. Om , S. D. Dhiman , D. P. Nandal , Indian J. Agron. 2006, 51, 77.

[37] Y. S. Shivay , R. Prasad , R. K. Singh , M. Pal , J. Agric. Sci. 2015, 7, 161.

[38] Y. S. Shivay , R. Prasad , J. Plant Nutr. 2012, 35, 928.

[39] R. Mahendra Kumar , V. Ravindra Babu , V. Subha Rao , K. Surekha , Ch. Padmavathi , M. S. Prasad , N. Somasekhar , A. S. Rama Prasad , V. Vinod Goud , Rice 2015, 8, 28.26362328

[40] C. M. Sunil , B. C. Shankaralingappa , M. K. Shruthi , S. Hittalmani , K. M. Harinikumar , J. Agron. 2014, 13, 58.

[41] A. K. Thakur , S. Rath , D. U. Patil , A. Kumar , Paddy Water Environ. 2011, 9, 13.

[42] L. Zhao , L. Wu , Y. Li , X. Lu , D. Zhu , N. Uphoff , Exp. Agric. 2009, 45, 275.

[43] P. Suryavanshi , Y. V. Singh , R. Prasanna , A. Bhatia , Y. S. Shivay , Paddy Water Environ. 2013, 11, 321.

[44] M. A. Gomma , F. I. Radwan , E. E. Kandil , M. A. M. Shawer , Middle East J. Agric. Res. 2015, 4, 919.

[45] S. Gopalakrishnan , R. Mahender Kumar , P. Humayun , V. Srinivas , B. Ratna Kumari , R. Vijayabharathi , A. Singh , K. Surekha , Ch. Padmavathi , N. Somashekar , P. Raghuveer Rao , P. C. Latha , L. V. Subba Rao , R. V. Babu , B. C. Viraktamath , V. Vinod Goud , N. Loganandhan , B. Gujja , O. Rupela , Paddy Water Environ. 2014, 12, 79.

[46] R. Adusumilli , S. B. Laxmi , Paddy Water Environ. 2011, 9, 89.

[47] Y. S. Shivay , R. Prasad , M. P. Singh , J. Plant Nutr. 2016, 39, 875.

[48] M. A. Ali , J. K. Ladha , J. Rickman , J. S. Lales , J. Crop Improv. 2006, 16, 173.

[49] A. Satyanarayana , T. M. Thiyagarajan , N. Uphoff , Irrig. Sci. 2006, 25, 99.

[50] A. J. Walkley , I. A. Black , Soil Sci. 1934, 37, 29.

[51] B. V. Subbiah , G. L. Asija , Curr. Sci. 1956, 25, 259.

[52] R. Olsen , C. V. Cole , F. S. Watanabe , L. A. Dean , Estimation of Available Phosphorus in Soils by Extraction with Sodium Bicarbonate: Circular‐939, United States Department of Agriculture, Washington, DC 1954.

[53] J. J. Hanway , H. Heidel , Soil Analysis Methods as Used in Iowa State College Soil Testing Laboratory: Bulletin 57, Iowa State College of Agriculture, Iowa, USA 1952, p. 131.

[54] W. L. Lindsay , W. A. Norvell , Soil Sci. Soc. Am. J. 1978, 42, 421.

[55] A. K. Singh , V. Chinnusamy , Indian Farming 2007, 57, 7.

[56] T. Satake , Modern Rice Milling Technology, University of Tokyo Press, Tokyo, Japan 1990.

[57] K. A. Gomez , A. A. Gomez , Statistical Procedures for Agricultural Research, 2nd ed., John Wiley and Sons, New York 1984, p. 680.

